# Rhythmic TMS over Parietal Cortex Links Distinct Brain Frequencies to Global versus Local Visual Processing

**DOI:** 10.1016/j.cub.2011.01.035

**Published:** 2011-02-22

**Authors:** Vincenzo Romei, Jon Driver, Philippe G. Schyns, Gregor Thut

**Affiliations:** 1 Centre for Cognitive Neuroimaging, Institute of Neuroscience and Psychology, University of Glasgow, Glasgow G12 8QB, UK; 2 Wellcome Trust Centre for Neuroimaging at UCL, Institute of Neurology, University College London, London WC1N 3BG, UK; 3 UCL Institute of Cognitive Neuroscience, University College London, London WC1 3AR, UK

## Abstract

Neural networks underlying visual perception exhibit oscillations at different frequencies (e.g., [1–6]). But how these map onto distinct aspects of visual perception remains elusive. Recent electroencephalography data indicate that theta or beta frequencies at parietal sensors increase in amplitude when conscious perception is dominated by global or local features, respectively, of a reversible visual stimulus [6]. But this provides only correlative, noninterventional evidence. Here we show via transcranial magnetic stimulation (TMS) interventions that short rhythmic bursts of right-parietal TMS at theta or beta frequency can causally benefit processing of global or local levels, respectively, for hierarchical visual stimuli, especially in the context of salient incongruent distractors. This double dissociation between theta and beta TMS reveals distinct causal roles for particular frequencies in processing global versus local visual features.

## Graphical abstract

**Graphical Abstract:**

**Figure:**
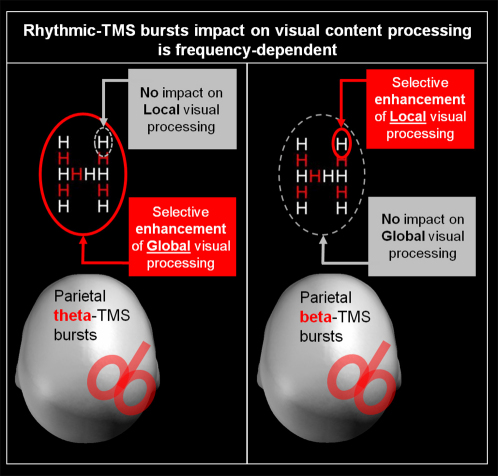

**Highlights:**

► Rhythmic TMS causally targets particular brain processes in a frequency-specific way ► Right-parietal TMS at beta-frequency selectively enhances local visual processing ► Right-parietal TMS at theta-frequency selectively enhances global visual processing ► Rhythmic TMS is a powerful tool for frequency-specific interventions in brain function

## Results and Discussion

We tested whether theta and beta rhythms are causally related to global versus local visual processing via the emerging method of “rhythmic” transcranial magnetic stimulation (TMS). This stimulates the brain with short bursts of TMS at particular frequencies [7–10] to test any causal relation to specific processes. We tested for a double dissociation between the impact of TMS bursts at theta versus beta frequency on global versus local visual processing.

**Figure 1:**
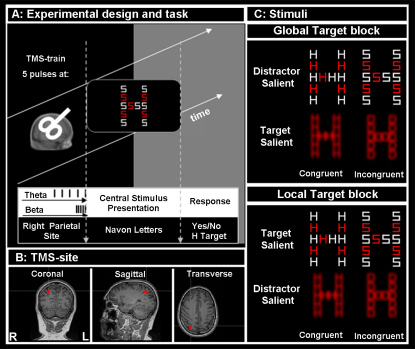
Experimental Design, Task, Stimuli, and Stimulation Site (A) Experimental design and task. Rhythmic TMS was applied in short bursts of five pulses at one of two frequencies (theta band, 5 Hz; beta band, 20 Hz) on each trial in the main experiment, in random order across trials, with 10 s intervening between successive bursts. Onset of a global/local hierarchical visual stimulus, centered at fixation, coincided with the last TMS pulse of each burst so that the critical visual display closely followed the rhythmic TMS bursts (future studies might vary this timing to examine the temporal profile of our effects). A sham TMS condition was also conducted (coil tilted at 90° over the same parietal site), in separate blocks that were randomly intermingled with active-TMS blocks. (B) Stimulation site for one representative participant. TMS was applied over a right-hemisphere intraparietal sulcus site in our main experiment, determined by neuronavigation with Brainsight and individual anatomical MRI scans, at Tailarach coordinates 28, −51, 50 (see Results and Discussion and Experimental Procedures). (C) Examples of stimuli for the global/local target blocks. In the global target blocks, observers were asked to detect the presence (versus absence) of the global letter H (versus S or D). The local distractors were all Hs, or all Ss or Ds, independent of global identity, leading to equiprobable congruent and incongruent conditions. For blurred stimuli, the global letter was more salient than the local letters; the reverse was true for nonblurred stimuli. In other blocks of trials, the same stimuli were used, but the local level was judged instead. For both tasks, salient incongruent distractors interfered the most (see Supplemental Experimental Procedures and Figure S1).

We applied TMS bursts at either frequency over a right parietal site (see Figure 1B and Experimental Procedures) immediately prior to visual stimulus onset (Figure 1A). Our global/local stimuli comprised Navon-like hierarchical letters [11], adapted from [12] (see Experimental Procedures). The stimuli were blurred or unblurred, making the global or local level, respectively, more salient (see Figure 1C). Participants judged the presence or absence of a target letter at the global level while ignoring the local level, or vice versa, with attended level blocked. Depending on stimulus format and which level (global or local) was judged, either the target level was more salient than the distractor level or vice versa (see Figure 1C). The letter or letters at the distractor level were congruent or incongruent with the letter or letters at the target level. Instructions stressed both speed and accuracy. For simplicity, we focus on one score that combines reaction time and accuracy (inverse efficiency, which is reaction time divided by proportion correct [12, 13]), but our critical results were present for all these measures.

A sham-TMS control (see Experimental Procedures) served to establish baselines in separate blocks from active TMS. The sham conditions replicated previous behavioral findings [12] of stronger interference from incongruent distractors at the currently irrelevant level when these were more salient (see Figure S1A available online). These sham-TMS conditions control for nonspecific effects, such as the “click” sounds made by TMS bursts at different frequencies (see Supplemental Experimental Procedures). We then subtracted sham TMS from active TMS for each condition and frequency to test whether active TMS at different frequencies would modulate the striking interference found from salient incongruent distractors in the sham conditions. We hypothesized (based on existing electroencephalography [EEG] data [6]) that beta TMS would benefit local targets, thereby reducing interference from salient global incongruent distractors, whereas theta TMS would benefit global targets, thereby reducing interference from salient local incongruent distractors.

Active versus sham rhythmic TMS did affect processing of local and global levels differentially in a manner that depended, as anticipated, on TMS frequency (interaction of task × TMS frequency in repeated-measures analysis of variance [ANOVA] on active − sham differences; F(1,11) = 9.1, p = 0.01). Active theta TMS (but not beta) enhanced performance for the global task (theta TMS versus sham, improvement of −35 for inverse efficiency ± 17.6 standard error, t(11) = −3.76, p = 0.003), whereas for active beta TMS versus sham, any nonsignificant trend in the global task was for impaired performance instead (+13.3 ± 26.8, t(11) = 0.65, not significant [NS]). Conversely, active beta TMS (unlike theta) versus sham enhanced performance for the local task instead (improvement of −59.34 ± 40.6, t(11) = −3.5, p = 0.005), whereas for active theta TMS versus sham, any nonsignificant trend in the local task was for worse performance instead (+16.5 ± 30.3, t(11) = 0.99, NS). This indicates a differential role of theta and beta rhythms for global versus local tasks, respectively.

**Figure 2:**
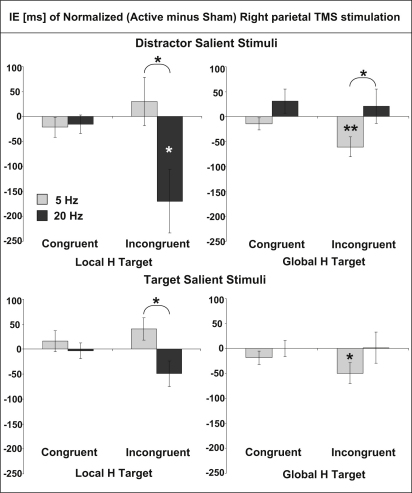
Effects of Rhythmic TMS on Performance for Global or Local Target Identification Sham-normalized (active − sham TMS at each frequency) effects of rhythmic right-parietal TMS bursts at 5 Hz (light gray bars) or 20 Hz (dark gray bars) in the global task (right) or the local task (left). Data are shown separately for distractor-salient and target-salient conditions; these show a similar pattern that is stronger with salient distractors (top). The y axis plots mean differences (for active − sham) in inverse efficiency (±standard error of the mean), so that negative values correspond to improved performance with active TMS and positive values correspond to impaired performance with active TMS. Asterisks indicate significant differences on t tests, either from the null hypothesis of no difference between active and sham TMS or between pairs of conditions as bracketed. ^∗^p < 0.05; ^∗∗^p < 0.01. See also Supplemental Experimental Procedures, Figure S2, and Table S1.

As anticipated, these frequency-specific benefits mainly arose for trials with interfering incongruent rather than congruent distractors, leading to a task × TMS frequency × incongruency interaction (F(1,11) = 11.2, p = 0.007). Effects of theta TMS versus sham for global targets were significant for incongruent (t(11) = −3.09, p = 0.01) but not congruent (t(11) = −1.82, NS) trials; likewise, effects of beta TMS for local targets were significant for incongruent (t(11) = −3.15, p = 0.009) but not congruent (t(11) = −0.67, NS) trials. Finally, the benefits from the appropriate TMS frequency for a given target level were particularly pronounced for distractor salient incongruent trials (leading to an interaction of task, TMS frequency, congruency, and saliency: F(1,11) = 7.84, p = 0.017). These distractor salient incongruent trials are those that led to the most interference in the absence of TMS (as in [12]), as we replicated here for the sham conditions (see Figure S1A). Correspondingly, these trials benefit most from TMS at the appropriate frequency (see Supplemental Experimental Procedures). Figure 2 plots sham-normalized changes in inverse efficiency due to TMS at one or the other frequency for each condition. Lower (negative) scores correspond to TMS-enhanced performance; higher scores correspond to worse performance. Note that beta TMS particularly enhances performance for the local task with incongruent salient distractors, whereas theta TMS particularly enhances performance for the global task with incongruent salient distractors. This same pattern is also evident for the RT or accuracy measures when analyzed alone in the same way (see Supplemental Experimental Procedures and Tables S1A and S1B). A similar but reduced pattern is seen for target-salient conditions.

Our key finding is the double dissociation in how TMS at theta or beta frequency impacts on global or local tasks, respectively. Although some previous work has shown that rhythmic TMS can be more effective at one frequency than another (e.g., for some interesting visual effects with TMS at alpha [8–10]), the present study, to our knowledge, is the first to show that one task can be affected by a particular frequency but not the other, whereas the reverse holds for another task. Our effects are therefore genuinely frequency specific, rather than having one particular frequency being more powerful overall. These results show that directly stimulating parietal cortex at perceptually relevant frequencies, via rhythmic TMS, can be used to bias visual processing toward one or the other of the two competing levels of hierarchical figures. This provides new causal TMS evidence for a relationship between particular frequencies and perception of the local or global properties of an image.

At the request of reviewers, we performed two additional control experiments to confirm the specificity of our results. In the first control, we applied TMS over left rather than right parietal cortex. This had no systematic impact on performance (see Figure S2A) and differed significantly (see Supplemental Experimental Procedures) from our critical findings for right-parietal TMS in our main experiment. In the second control, we stimulated the right parietal site at 10 Hz (because 10 Hz is a harmonic of 5 Hz, and activity in the alpha-band at around 10 Hz is known to be important for visual processing; e.g., [2–5, 8–10]). The outcome for 10 Hz differed qualitatively and significantly from 5 Hz TMS (see Supplemental Experimental Procedures and Figure S2B), confirming that our key finding was indeed specific to 5 Hz stimulation and was not observed with higher harmonics. Thus, the present results are specific to the frequency of rhythmic TMS and to the right rather than left parietal site, though it would be useful to examine further right-hemisphere sites in future extensions.

Returning to our main experiment, the brief online rhythmic TMS bursts did not impair but rather enhanced performance in a frequency-dependent way. This does not accord with the traditional concept of TMS acting as a “virtual lesion” but fits other evidence showing that some forms of TMS can enhance performance [7–10]. One interpretation of our current right-parietal results at 5 Hz and 20 Hz could be in terms of recent proposals [7–10] stating that rhythmic TMS bursts may impact ongoing oscillatory neural activity. In principle, this might even involve entrainment of neural networks oscillating at the TMS frequency, which future electrophysiological studies could test. It has been suggested that theta may provide an important resonance frequency for brain networks involved in binding global information together, whereas more local networks may oscillate at higher frequencies (e.g., [14]).

Any cross-frequency “rivalry” between theta and beta appears to be less likely to explain our TMS effects, because right-parietal theta TMS facilitated the global task but did not impair the local task, whereas the opposite was true for beta TMS. Refining the exact role of particular brain oscillations will ultimately require neurophysiological measures (e.g., EEG), as well as rhythmic TMS interventions. Indeed, it should be particularly interesting to combine rhythmic TMS with online neural measures in future work. However, our behavioral results already demonstrate a doubly dissociated impact of beta versus theta TMS for the first time. Rather than one TMS frequency being most effective overall, beta TMS had the most impact on the local task, whereas theta TMS had the most impact on the global task. In providing this double dissociation, our results illustrate more generally the potential of rhythmic TMS for frequency-specific interventions in brain function.

## Experimental Procedures

### Main Experiment

#### Participants

All 12 healthy volunteers had normal or corrected vision by self-report (mean age 28.25 yr, range 20–42 yr, five females). All gave written informed consent in accord with local ethical approval (Faculty of Information and Mathematical Sciences, University of Glasgow) and were right handed by self-report.

#### Visual Stimuli and Task

These aspects were adapted from a previous study [12]. Stimuli were presented on a 17 inch monitor (85 Hz refresh rate) on a black background. Viewing distance was approximately 60 cm. White central dots indicated fixation, appearing for 1.5 s followed by 200 ms of blank screen to alert participants to the upcoming visual stimulus. Each such stimulus comprised a modified version of Navon hierarchical letters [11], centered at the fixation point, using the same modifications to these as in [12].

For the nonblurred displays with relatively high local saliency, the Navon stimuli were created from orthogonal combinations of the letters H or S at the global level, and multiple Hs or Ss at the local levels, with the letters in the local dimension alternating between red and white (see Figure 1A). Each local letter subtended approximately 1.34° × 1.06° of visual angle (in height and width, respectively), and the global letter subtended 8.26° × 5.38° of visual angle with an interlocal-letter distance of 0.96°.

For the blurred displays with relatively high global saliency, the Navon stimuli were created from orthogonal combinations of the letters H and D, but now all local letters were red and underwent a blurring procedure in Paint Shop Pro 7.0 with factor = 7 (see Figure 1A). Each local letter again subtended ∼1.34° × 1.06° of visual angle (in height and width, respectively), and the global letter subtended 5.66° × 4.51° of visual angle with an interlocal-letter distance of 0.38°. For further stimulus details, see [12].

Participants were instructed to detect the presence of a target H, or its absence (S or D instead), at either the local or global level while ignoring the other level, with this instruction applying for a block of trials and with task blocks in random order. Participants responded on a two-choice button box with right index finger for target presence and right middle finger for target absence.

#### Experimental Procedure

Rhythmic TMS was applied while participants performed the Navon letter discrimination task at the currently task-relevant level. Rhythmic TMS was administered in short bursts of five pulses at one of two different frequencies on each trial (5 Hz versus 20 Hz, random order across trials), immediately prior to visual presentation (target onset synchronized with fifth TMS pulse; see Figure 1A). Trials, and thus TMS bursts, were separated by 10 s. The experiment comprised six blocks per condition, for active or sham TMS, in separate blocks of 32 trials (≈6–7 min per block), resulting in a total of 960 active TMS pulses (plus 960 sham pulses).

#### TMS Stimulation

TMS was applied at a fixed intensity level of 60% of maximum stimulator output using a Magstim Rapid^2^ Transcranial Magnetic Stimulator via a 70 mm figure-of-eight coil (Magstim Company). In our main experiment, the TMS site was over the right intraparietal sulcus (Talairach coordinates: 28, −51, 50). This site has previously been shown with fMRI to be coactivated across several attention tasks, including feature-based attention [15]. We neuronavigated the TMS coil to this target site in each individual via Brainsight (Rogue Research) in combination with individual structural MRI scans that could be normalized into standard Tailairach space to identify particular coordinates and then back into native space.

During active TMS, the coil handle was oriented parallel to the sagittal plane and then tilted forward or backward until the coil surface was tangential to the scalp. Most of the current was therefore induced in the anterior-posterior (y axis) and superior-inferior (z axis) dimension, with only minor contribution to the left-right dimension (x axis). Sham stimulation (coil tilted at 90° over the same area as active TMS; see also [8–10, 16, 17]) was performed to account for any nonspecific effects of TMS due to the associated auditory clicks. The rate of these clicks over time (5 Hz or 20 Hz) inevitably covaried with TMS frequency, hence the critical importance of sham controlling the impact of active TMS (see also Supplemental Experimental Procedures).

The experiment comprised active and sham TMS blocks that were intermingled across the experimental session and counterbalanced across participants. TMS frequency (5 Hz or 20 Hz) was intermingled in an event-related manner. Note that this will inherently subtract out any frequency-dependent carry-over effects from one trial to the next because of the randomized event-related ordering of conditions.

#### Data Analysis

To evaluate the within-trial effects of short bursts of rhythmic TMS on target discrimination at different frequencies, we subjected data for active − sham inverse efficiency scores to a four-way, repeated-measures ANOVA, with factors of TMS frequency (5 Hz versus 20 Hz), task (global versus local), incongruency (incongruent versus congruent displays), and distractor saliency (distractor salient versus target salient). Pairwise follow-up t tests were calculated when appropriate. See Supplemental Experimental Procedures for analyses performed on the sham-uncorrected data alone or on the active TMS data alone, as well as separately for RT and accuracy scores rather than for the combined inverse efficiency score.

For control experiments, see Supplemental Experimental Procedures.

## Supplemental Information

Document S1. Supplemental Experimental Procedures, One Table, and Three Figures
