# Supplementary material for: Rhythmic TMS over Parietal Cortex Links Distinct Brain Frequencies to Global versus Local Visual Processing
Source: Curr Biol. 2011 Feb 22;21(4):334–7. doi: 10.1016/j.cub.2011.01.035 (PMC3063337; doi:10.1016/j.cub.2011.01.035)
Supplement: Document S1. Supplemental Experimental Procedures, One Table, and Three Figures [file mmc1.pdf]

Current Biology, Volume *21*

Supplemental Information

Rhythmic TMS over Right Parietal Cortex Causally Links Distinct Brain Frequencies to Global versus Local Visual Processing

Vincenzo Romei, Jon Driver, Philippe Schyns, and Gregor Thut

Supplemental Data

Analysis of Right-Parietal Sham Data Only

For completeness we ran a similar four-way ANOVA as in the main paper (task x incongruency x distractor saliency x TMS frequency) but on the sham data alone from the right-parietal experiment. This showed the expected impacts of task, incongruency and saliency, but no interactions involving TMS frequency. Performance was better in the global than local task overall (mean inverse efficiency of 533 vs 612, F(1,11)=18.4; p=0.001), in the congruent versus incongruent conditions (519 vs 626, F(1,11=26.0; p=0.0003), and with target rather than distractor salient (526 vs 619, F(1,11=18.3; p=0.001). The distractor saliency effect was somewhat larger in the local task (interaction of task and saliency, F(1,11)=13.6, p=0.004); as was the congruency effect (interaction between task and congruency, F(1,11)=5.9, p=0.03); see Supplemental Fig. S1a. Importantly there were no significant interactions involving TMS frequency (all p>0.12), just a marginal tendency for better performance with 5Hz than 20Hz sham overall (557 vs 588 mean inverse efficiency, F(1,11)=4.1, p=0.07), presumably due to the higher rate of associated ‘click’ sound being somewhat more distracting. Once again this underlines the importance of sham-controlling the active-TMS data, as per all the analyses in the main paper.

Analysis of Right-Parietal Active-TMS Data Only

For completeness, we also analysed just the active-TMS data from the right-parietal experiment (although we note the importance of sham-controlling this, as per the main paper). We again found better performance in the global versus local task overall (mean inverse efficiency of 522 vs 597, F(1,11)=15.2, p=0.002), in congruent than incongruent conditions (516 vs 597, F(1,11)=52.8, p=0.00002) and with salient targets rather than salient distractors (518 vs 594, F(1,11)=22.1, p=0.0006). The interactions between task and saliency (F(1,11)=7.0, p=0.02) and between task and congruency (F(1,11)=5.9, p<0.03) were also as before. Unlike the sham data we found that active TMS at 5Hz vs 20Hz influenced performance differentially in the global and local task (significant interaction between task and frequency, F(1,11)=6.3, p<0.03), with the global task better under 5Hz than 20Hz (491 vs 553 mean inverse efficiency), but vice-versa for the local-task (603 vs 578).

RT and Accuracy Data Considered Separately for the Main Right-Parietal TMS Experiment

A reviewer asked that we provide separate RT and accuracy results, in addition to the combined inverse efficiency (IE) measure presented in the main text, which we had used in accord with a previous study that employed the same task [13] and with many other prior studies e.g.[16-19]. Supplemental Table S1a lists mean RTs per condition; while Supplemental Table S1b lists the corresponding error-rates. These RTs and error-rates were analysed in exactly the same way as for the IE scores in the main paper. Note that interpretation of our critical conditions was not complicated by any problematic speed/error tradeoffs. The critical effects of right-parietal TMS reported for the combined IE scores in the main text (as summarised in main Fig 2) were also present for RT considered alone, or for accuracy considered alone. Thus performance was both faster ($p<0.02$) and more accurate ($p<0.02$) in the global task, with incongruent salient local distractors, during active versus sham 5Hz right-parietal TMS. Likewise, performance was both faster ($p<0.05$) and more accurate ($p<0.01$) in the local task, with incongruent salient global distractors, during active versus sham 20Hz right-parietal TMS.

Manipulation of the Relative Salience of Global and Local Levels

Some previous work studying lesioned patients [e.g. 1,2], or using PET/fMRI [3-6] or EEG methods [7-10] in normals, was taken to indicate that the right-hemisphere may be preferentially involved in global processing, but this is debated (e.g. [11,12]). Moreover the relative salience of global and local levels was typically not manipulated in such work (though see [13,14]), making it hard to determine whether global/local level or instead relative salience was critical in those previous studies. Here in keeping with recent work [13,14] we varied which level was more salient, orthogonally to which level was judged. It has been claimed that inactivation (with prolonged off-line repetitive TMS, unlike the brief on-line bursts used here) of right parietal cortex can disrupt the impact of more salient stimuli [13,14]. Here using a very different TMS protocol with short rhythmic bursts, we found that right-parietal TMS could reduce interference from salient distractors. But critically the level (global or local) which benefited from the TMS bursts here depended on right-parietal TMS frequency, which was not considered in previous work. We found that the local task benefited from beta-TMS bursts, while the global task benefited from theta-TMS bursts instead, at the same right parietal stimulation site, in keeping with the EEG results [15] that had led to our hypothesis.

First Control Experiment with Left-Parietal TMS at 5 and 20Hz

At the request of reviewers, we repeated the full design of the main experiment in 12 new participants (mean age 27.1, range 19-36, 8 female, recruited at University College London in accord with local ethics), but now using TMS over the homologous left parietal site instead (MNI coordinates of -28, -51, 50). The left-parietal sham results did not differ from the right-parietal sham results as a function of task condition or sham frequency (compare Figs S1a and S1b). The sham-controlled (active-minus-sham) TMS results within the new left-parietal experiment are shown in Supplemental Fig S2a, in the same format as shown for the right-parietal experiment in main Fig 2. Unlike the right-parietal data, there are no systematic TMS

effects on performance in the new left-parietal control experiment. In a comparable four-way ANOVA on the left-parietal IE data, there were no significant terms involving TMS frequency (all $p>0.11$). Moreover the new outcome for left-parietal TMS differed significantly from that for the original right-parietal TMS; at $p<0.02$ for the 5Hz impact on the global task with salient incongruent local distractors; and at $p<0.03$ for the 20Hz impact on the local task with salient incongruent global distractors, in unpaired t-tests.

Second Control Experiment with Right-Parietal TMS at 10Hz

At the request of one reviewer, we also performed a further control experiment (with 11 subjects from UCL, mean age 24.4, range 19-35, 6 female), using the original right-parietal site with the same task, but now stimulating only at 10Hz. The reviewer had noted that 10Hz is a harmonic of 5Hz, so wondered if the critical impact for the global task might actually be in the alpha rather than theta range. The sham-controlled (active-minus-sham) results within the new 10Hz right-parietal experiment are shown in Supplemental Fig S2b. The pattern of results clearly differs qualitatively to that originally found for right-parietal 5Hz (compare Supplemental Fig S2b with light bars in main Fig 2). The enhanced performance found with 5Hz for the global task with incongruent salient local distractors was absent with 10Hz (significant difference between 5Hz and 10Hz data, $p<0.04$). 10Hz produced some distinctive effects of its own (notably impaired performance for the global task with congruent salient distractors, but enhanced for the local task with congruent salient distractors, see Supplemental Fig S2b). Understanding these particular effects of right-parietal 10Hz TMS will require further research, but for present purposes these control results add further confirmation to the specificity of our main results, showing that the impact of 5Hz TMS is not reproduced at 10Hz (nor at 20Hz, see main text).

Table S1 A.	RTs [ms] of Normalized (active minus sham) TMS stimulation from main experiment (related to Figure 2)							
	Local H Target				Global H Target			
	Congruent		Incongruent		Congruent		Incongruent	
	5Hz	20Hz	5Hz	20Hz	5Hz	20Hz	5Hz	20Hz
Distractor Salient Stimuli	**-17.96 (±17.83)**	**-3.71 (±16.17)**	**13.34 (±21.15)**	**-12.02 (±7.20)**	**-7.26 (±12.21)**	**44.42 (±22.41)**	**-24.97 (±10.63)**	**18.46 (±22.45)**
Target Salient Stimuli	**-0.24 (±17.66)**	**-4.37 (±12.80)**	**24.19 (±22.07)**	**-47.60 (±16.15)**	**-16.40 (±10.67)**	**-9.31 (±13.98)**	**-47.83 (±19.83)**	**5.92 (±18.68)**

Table S1 B.	Error rate [%] of Normalized (active minus sham) TMS stimulation from main experiment (related to Figure 2)							
	Local H Target				Global H Target			
	Congruent		Incongruent		Congruent		Incongruent	
	5Hz	20Hz	5Hz	20Hz	5Hz	20Hz	5Hz	20Hz
Distractor Salient Stimuli	**0 (±1.86)**	**-2.08 (±1.14)**	**0 (±3.39)**	**-10.42 (±3.41)**	**0 (±1.51)**	**-2.08 (±1.56)**	**-5.56 (±2.47)**	**2.08 (±2.24)**
Target Salient Stimuli	**2.78 (±1.24)**	**2.78 (±1.24)**	**0 (±1.51)**	**0 (±2.62)**	**0 (±1.07)**	**1.39 (±1.45)**	**0 (±1.51)**	**-.069 (±1.99)**

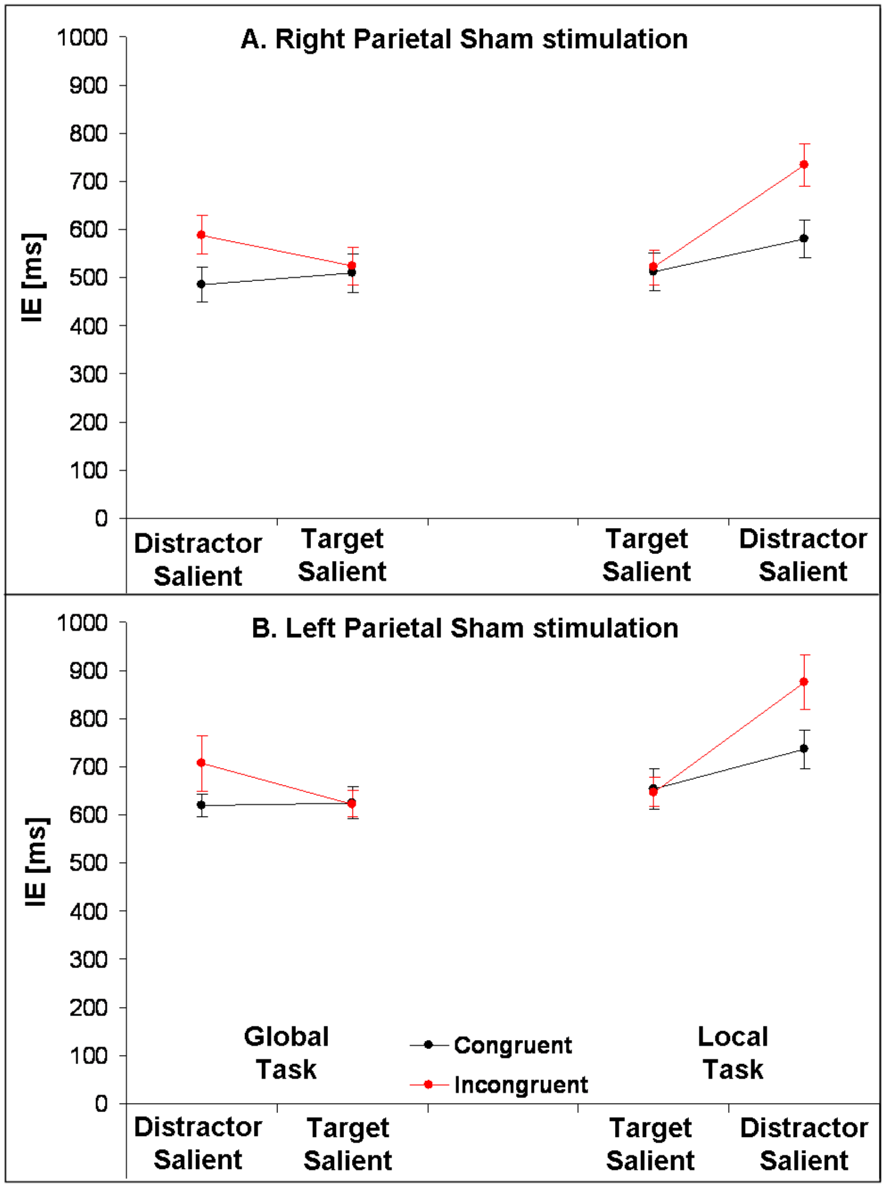

Figure S1, Related to Figure 1.

Baseline performance (sham blocks only) for global or local target identification as a function of congruency and saliency in: (a) the right-parietal 5/20Hz main experiment and (b) the left-parietal 5/20Hz control experiment. The mean inverse efficiency scores (RTs divided by proportion correct), ± s.e.m., are shown, for identifying global or local targets as a function of distractor congruency (congruent trials, black line; incongruent trials, red line) and distractor saliency (Distractor or Target Salient) in the sham blocks. As expected, performance is worse (higher scores) for incongruent trials than congruent, especially when the distractor is salient [13].

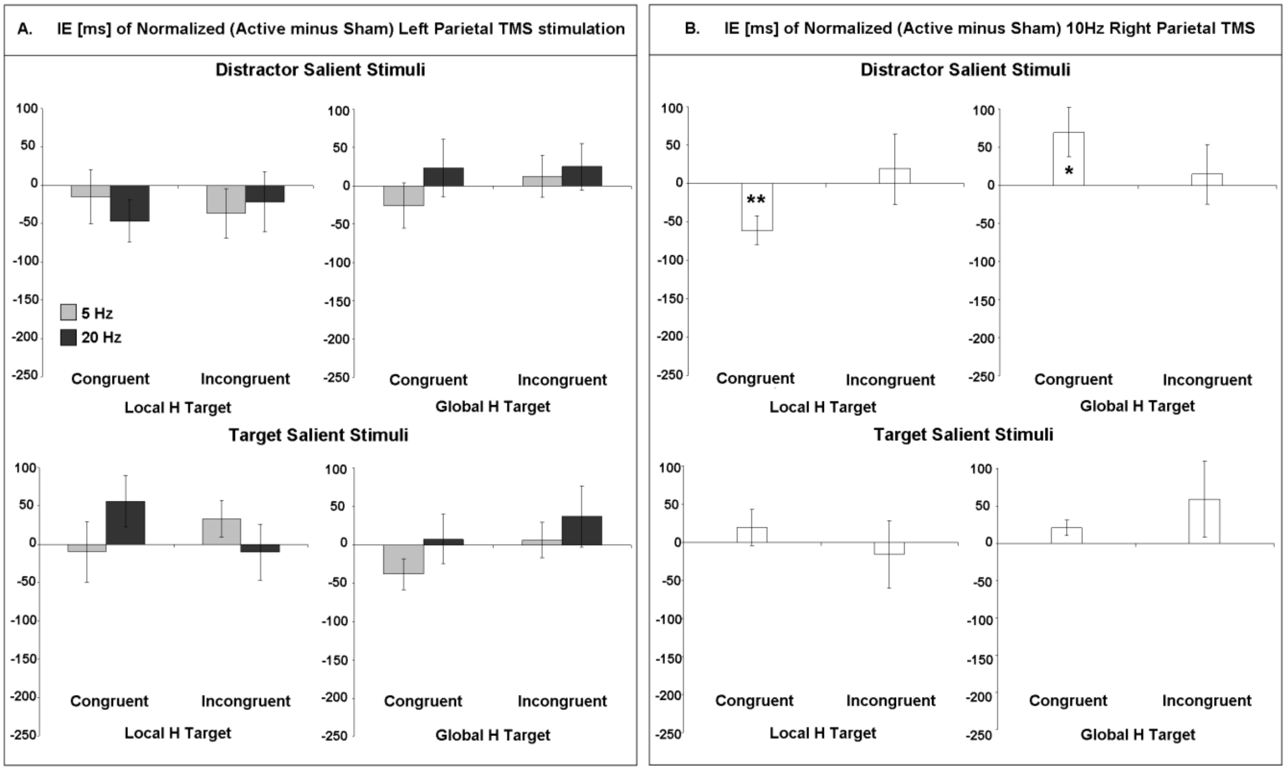

Figure S2, Related to Figure 2.
A. Left-parietal rhythmic TMS at 5Hz or 20Hz has no impact on performance for global or local target identification. Sham-normalized (active minus sham TMS at each frequency) results for left-parietal TMS bursts at 5Hz (light-gray bars) or 20Hz (dark-gray) in the global task (right panels) or the local (left panels) task. Upper panels are for distractor-salient conditions, lower panels for target-salient. The plot is analogous to the plot for the right-parietal 5/20Hz experiment shown in main Figure 2. The y-axis plots mean differences (for active minus sham) in inverse efficiency (± s.e.m.). Active left-parietal TMS produced no significant differences from sham. Moreover this outcome was significantly different to the systematic effects found with right-parietal TMS instead; see text.
B. Right-parietal rhythmic TMS at 10Hz has a qualitatively different impact on performance than the original right-parietal 5Hz effect. Sham-normalized (active minus sham TMS) results for right-parietal TMS bursts at 10Hz in the global task (right panels) or the local (left panels) task. Upper panels are for distractor-salient conditions, lower panels for target-salient. The y-axis plots mean differences (for active minus sham) in inverse efficiency (± s.e.m.). The pattern is qualitatively different to that found for right-parietal 5Hz TMS (compare with light bars in main Fig 2), and differed significantly from that (see text).
Within the new 10Hz experiment, the only significant TMS effects now applied to congruent rather than incongruent conditions (with salient distractors), and led to an impairment in the global task but a benefit in the local task for the same 10Hz frequency. Further understanding of the 10Hz impact will require more research. For present purposes it serves as a control to further underline the frequency-specificity of the 5Hz and 20Hz right-parietal effects; see text.

Supplemental References

[1] Robertson, L.C., Lamb, M.R. and Knight, R.T. (1988). Effects of lesions of temporal-parietal junction on perceptual and attentional processing in humans. J Neurosci. 8, 3757-69.
[2] Lamb, M. R., Robertson, L. C., and Knight, R. T. (1990). Component mechanisms underlying the processing of hierarchically organized patterns: inferences from patients with unilateral cortical lesions. J. Exp. Psychol. Learn. Mem. Cogn. 16, 471-483.
[3] Martinez, A., Moses, P., Frank, L., Buxton, R., Wong, E. and Stiles J. (1997). Hemispheric asymmetries in global and local processing: evidence from fMRI. Neuroreport, 8, 1685-1689.
[4] Fink, G.R., Halligan, P.W., Marshall, J.C., Frith, C.D., Frackowiak, R.S. and Dolan, R.J. (1996). Where in the brain does visual attention select the forest and the trees? Nature, 382,626-628.
[5] Fink. G,R., Marshall, J.C., Halligan, P.W., Frith, C.D., Frackowiak, R.S. and Dolan, R.J. (1997). Hemispheric specialization for global and local processing: the effect of stimulus category.Proc. Biol. Sci. 264, 487-494.
[6] Fink, G.R., Halligan, P.W., Marshall, J.C., Frith, C.D., Frackowiak, R.S. and Dolan, R.J. (1997). Neural mechanisms involved in the processing of global and local aspects of hierarchically organized visual stimuli. Brain, 120, 1779-1791.
[7] Heinze, H. J. and Munte, T. F. (1993). Electrophysiological correlates of hierarchical stimulus processing: dissociation between onset and later stages of global and local target processing. Neuropsychologia, 31, 841–852.
[8] Proverbio, A. M., Minniti, A., and Zani, A. (1998). Electrophysiological evidence of a perceptual precedence of global vs. local visual information. Brain Res. Cogn. Brain Res. 6, 321-334.
[9] Evans, M. A., Shedden, J. M., Hevenor, S. J., and Hahn, M. C. (2000). The effect of variability of unattended information on global and local processing: evidence for lateralization at early stages of processing. Neuropsychologia, 38, 225-239.
[10] Volberg, G., Kliegl, K., Hanslmayr, S. and Greenlee, M.W. (2009). EEG alpha oscillations in the preparation for global and local processing predict behavioral performance. Hum. Brain Mapp. 30, 2173-2183.
[11] Boles, D.B., and Karner, T.A. (1996). Hemispheric differences in global versus local processing: still unclear. Brain Cogn. 30, 232-243.
[12] Johannes, S., Wieringa, B.M., Matzke, M. and Münte, T.F. (1996). Hierarchical visual stimuli: electrophysiological evidence for separate left hemispheric global and local processing mechanisms in humans. Neurosci. Lett. 210, 111-114.
[13] Mevorach, C. Humphreys, G.W. and Shalev, L. (2006). Opposite biases in salience-based selection for the left and right posterior parietal cortex. Nat. Neuroscience, 9, 740-742.
[14] Hodsoll, J., Mevorach, C. and Humphreys, G.W. (2009). Driven to less distraction: rTMS of the right parietal cortex reduces attentional capture in visual search. Cereb. Cortex, 19, 106-114.
[15] Smith, M.L. Gosselin, F. and Schyns, P.G. (2006). Perceptual moments of conscious visual experience inferred from oscillatory brain activity. Proc. Natl. Acad. Sci. U.S.A. 103, 5626-5631.
[16] Townsend, J.T. and Ashby, F.G. (1978). Methods of modeling capacity in simple processing systems. In N.J. Castellan & F. Restle (Eds.), Cognitive theory (Vol. 3, pp. 199-239). Hillsdale, NJ: Erlbaum.
[17] Townsend, J.T. and Ashby, F.G. (1983). Stochastic modelling of elementary psychological processes. New York: Cambridge University Press.

[18] Kennett, S., Eimer, M., Spence, C. and Driver, J. (2001). Tactile-visual links in exogenous spatial attention under different postures: convergent evidence from psychophysics and ERPs. J Cogn Neurosci. 13, 462-478.
[19] Spence, C., Kingstone, A., Shore, D.I. and Gazzaniga, M.S. (2001). Representation of visuotactile space in the split brain. Psychol. Sci. 12, 90-93.
